# Clinical Phenotypes of Adult-Onset Still’s Disease: New Insights from Pathophysiology and Literature Findings

**DOI:** 10.3390/jcm10122633

**Published:** 2021-06-15

**Authors:** Stéphane Mitrovic, Bruno Fautrel

**Affiliations:** 1 Service de Rhumatologie, Hôpital Pitié-Salpêtrière, Sorbonne Université—APHP, 75013 Paris, France; stephane.mitrovic@aphp.fr; 2 Centre d’Etude et de Référence sur les Maladies AutoInflammatoires et les Amyloses (CEREMAIA), FAI2R Network, 75013 Paris, France; 3 Département de Médecine Interne, Institut Mutualiste Montsouris, 75014 Paris, France; 4 Institut d’Epidémiologie et de Santé Publique Pierre Louis, UMR S 1136, Equipe PEPITES, 75013 Paris, France

**Keywords:** adult-onset Still’s disease, systemic-onset juvenile idiopathic arthritis, spondyloarthritis, psoriatic arthritis, osteitis, innate immunity, autoinflammation, immunological disease continuum, phenotypes, neutrophil urticarial dermatosis

## Abstract

Adult-onset Still’s disease (AOSD) is a non-familial, polygenic systemic autoinflammatory disorder. It is traditionally characterized by four cardinal manifestations—spiking fever, an evanescent salmon-pink maculopapular rash, arthralgia or arthritis and a white-blood-cell count (WBC) ≥ 10,000/mm^3^, mainly neutrophilic polymorphonuclear cells (PMNs)—but many other manifestations and complications can be associated, making clinical expression very heterogeneous and diagnosis sometimes difficult. The AOSD course can be diverse and is currently impossible to predict. Several clinical phenotypes have been described, either on the basis of the evolution of symptoms over time (monocyclic, polycyclic and chronic evolution) or according to dominant clinical evolution (systemic and arthritis subtypes). However, these patterns are mainly based on case series and not on robust epidemiological studies. Furthermore, they have mainly been established a long time ago, before the era of the biological treatments. Thus, based on our personal experience and on recent advances in the understanding of disease pathogenesis, it appears interesting to reshuffle AOSD phenotypes, emphasizing the continuum between AOSD profiles and other systemic autoinflammatory disorders, eventually proposing a research agenda.

## 1. Introduction

Adult-onset Still’s disease (AOSD) is classically described as a non-familial, or sporadic, systemic autoinflammatory disorder (SAID) [1]. Its incidence is estimated at 0.16 to 0.4 per 100,000 persons [2,3], and the reported prevalence rates range from 1 to 34 cases per 1 million persons in the Japanese and the European populations [3]. It is traditionally characterized by four cardinal manifestations—spiking fever, an evanescent salmon-pink maculopapular rash, arthralgia or arthritis and a white-blood-cell count (WBC) ≥ 10,000/mm^3^, mainly neutrophilic polymorphonuclear cells (PMNs)—but many other manifestations and complications can be associated, making clinical expression very heterogeneous [2] and diagnosis sometimes difficult. Classification criteria can be of help to perform the latter [4,5]. The AOSD course can also be diverse and is currently impossible to predict [6]. Several phenotypes have been described; however, these phenotypes are mainly based on case series and not on robust epidemiological studies [7,8,9,10,11]. Furthermore, they have mainly been established a long time ago, before the era of the biological treatments. In the first part of this paper, we will describe the “typical” presentation of AOSD and the evolution courses described so far. In the second part, based on our personal experience and on recent advances in the understanding of disease pathogenesis, we will discuss why it appears interesting to reshuffle AOSD phenotypes, emphasizing the continuum between AOSD profiles and with other systemic autoinflammatory disorders.

## 2. Common and Rare Clinical Manifestations

Besides the cardinal manifestations listed above, many other frequent clinical manifestations or biological abnormalities can exist in AOSD [1,2] and are recalled in the second section of Table 1. AOSD can also be responsible for a number of serious and life-threatening complications [12] (last section of Table 1). The physician should be aware of these atypical forms because the intensity of the general signs or organ involvement often relegates the cardinal signs to the background. The diagnosis of AOSD is then difficult and often delayed, after many other diagnostic hypotheses have been excluded. 

After comprehensive investigations, no clinical sign or biological abnormality is specific to AOSD to ascertain diagnosis. The classification criteria may be helpful, but these criteria were developed for clinical research rather than for diagnosis. Two sets of criteria have been validated. The Yamaguchi criteria, published in 1992, are the most widely used [4] (Table 2). These criteria include exclusion criteria such as infections, malignancies and other rheumatic diseases. They should be used only after a broad diagnostic work-up, which is problematic in clinical practice. The Fautrel criteria have the advantage of including ferritin and glycosylated ferritin (GF) levels as diagnostic biomarkers and do not require exclusion criteria [5] (Table 2). In a 2018 validation study, both sets showed high sensitivity and specificity [13].

## 3. Evolution Courses

Classically, AOSD phenotypes and evolution courses have been described based on two different aspects: the evolution of symptoms over time (monocyclic, polycyclic and chronic) and/or the type of symptoms, i.e., systemic (recurrent fever syndrome) versus articular (febrile, potentially erosive polyarthritis).

### 3.1. Classification Based on the Evolution Temporality

**Figure 1:**
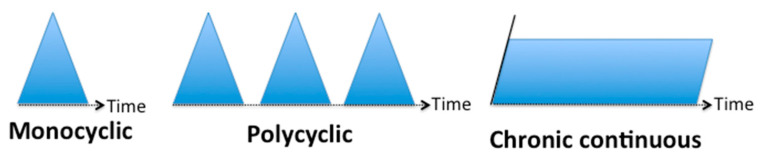
AOSD phenotypes based on the evolution of symptoms over time.

Historically, the clinical course of AOSD has been distinguished into three different phenotypes, described on the basis of the evolution of symptoms over time: monocyclic, polycyclic and chronic evolution (Figure 1) [1].

A monocyclic course is either self-limited or includes drug-free remission. The initial flare up with systemic manifestations and (potentially) joint involvement develops over a few weeks. The disease is limited to a single flare up and complete remission is achieved within a couple of weeks or months. Remission can be achieved with nonsteroidal inflammatory drugs, steroids or other immunomodulatory agents after a few days or weeks. These treatments can be progressively tapered then stopped without relapse after a few months. This phenotype seems to account for 19 to 44% of affected patients [9,10,14].

A recurrent or polycyclic course is characterized by AOSD relapse following either months or years of immunomodulatory treatment or therapy discontinuation. In this pattern, one possible presentation is a first flare up during systemic juvenile idiopathic arthritis (SJIA) diagnosed in childhood, followed by sustained drug-free remission for several years then a relapse in adulthood. In most of these cases, recurrences combine systemic manifestations and joint involvement. This phenotype represents 10 to 41% of affected patients [9,10,14].

Differently from the others, a chronic and progressive course involves continuous inflammation that is responsible for chronic erosive joint involvement with regular systemic flares. This phenotype is the most frequent, estimated at 35 to 67% of affected patients, in old series from the pre-biologic era [9,11,14,15]. 

### 3.2. Classification Based on the Type of Symptoms

In light of the new pieces of evidence about AOSD pathogenesis and treatment, especially the current use of biological therapy, this historic classification seems somehow outdated. 

Many authors have for some time now adopted a dichotomous classification, distinguishing two AOSD subtypes: systemic and articular. The systemic subtype includes patients with systemic features (such as high fever and skin rash), more at risk to develop life-threatening complications (such as multi-organ involvement and RHL). In the articular subtype patients have predominant articular involvement [16,17,18,19]. Classical articular manifestations described in AOSD are varied. Joint pain is the most common symptom, usually starting concomitantly with fever and with a maximal intensity during fever spikes. Arthritis is present in more than two-thirds of patients, migrating at the very beginning and becoming stable in the course of the disease. Bilateral symmetrical rheumatoid arthritis (RA)-like polyarthritis has been described. Any joint can be involved, more frequently radio-carpal or carpal joints (with the “classical bilateral ankylosing carpal arthritis”, which is very suggestive when “isolated”, i.e., without concomitant metacarpo-phalangeal or proximal interphalangeal structure damage) [1,2,9,11,15].

Predictive factors for the evolution towards each subset have been identified: high fever (>39 °C), hepatitis, thrombocytopenia, elevated CRP and hyperferritinemia seem to be associated with a systemic subset, while female gender, proximal arthritis at disease onset and steroid dependence are predictive of a chronic articular evolution [16,17,18,19,20,21]. 

### 3.3. Pathogenic Consideration and Expected Therapeutic Outcomes 

Substantial advances have been made to confirm the homology between AOSD and systemic-onset juvenile arthritis (SJIA, formerly called childhood-onset Still’s disease), and currently, most authors believe that they correspond to the same sporadic systemic auto-inflammatory disorder associated with inappropriate activation of the innate immune system at different ages [1]. It is thought to be a complex autoinflammatory interleukin (IL)-1-mediated disease, with IL-6- and IL-18-mediated inflammatory features as well. 

Data from the literature suggest that different cytokine profiles may be responsible for distinct clinical manifestations, as systemic subsets seem to present with high levels of IL-18 and IL-1β while patients with arthritis exhibit higher IL-6 serum levels [16,20]. In a cohort comparing 33 AOSD cases with 77 SJIA cases, patients with AOSD were classified into two subgroups based on serum IL-6 and IL-18 levels. The number of patients with arthritis was significantly higher in the IL-6-dominant subgroup, while no patient in the IL-18-dominant subgroup presented with arthritis [20]. However, these data should be treated with caution since “cytokine profiles” have not been clearly established. While IL-6 serum levels are higher in patients with arthritis, this cytokine has also been associated with severe systemic manifestations such as macrophage activation syndrome [17], and therapeutic blockade of IL-6 provided excellent clinical responses in SJIA patients with systemic phenotype [22]. 

Consequently, serum levels of cytokines are not routinely performed and do not highlight our daily management of individual patients. They remain to be validated in further prospective studies comparing “systemic” AOSD with “rheumatic” AOSD patients, in order to help “monitoring” the disease and eventually have an impact on therapeutic responses [6].

## 4. Why Reshuffling AOSD Phenotypes Is Needed

Limitations can be opposed to the different classifications described above and recent observations raise questions and remind us that the reality of things is complex, as it is often the case in medicine. 

First, the dichotomous “systemic/articular” classification is certainly interesting but probably too simplistic with unresolved points (if only the discordance of the cytokine profiles reported above). Then, the historical description of the evolution of profiles according to symptoms over time is based on case series and not on robust epidemiological studies. Many articular descriptions in these historic cohorts predate the establishment of the Yamaguchi criteria [9,11,14,15]. Thus, it cannot be clearly identified if all the patients included actually correspond to AOSD or to another category of rheumatism, for instance, a febrile-onset RA. It is possible that some patients with RA-like polyarthritis, classified at that time as AOSD, would nowadays be classified as a seronegative rheumatoid arthritis [23]. Furthermore, the “chronic continuous”’ profile was the most frequent in old series from the pre-biologic era. No new estimates are available in adults, but the frequency of this specific profile is likely to be low (if still existing) at the era of biological therapies, especially given the observations of our pediatrician colleagues over the past years in patients with systemic onset juvenile arthritis (SJIA).

### 4.1. The Contribution of Research on SJIA: The Concepts of Window of Opportunity, Treat-to-Target and Phenotype Change

Interestingly, observations in several case series and one prospective cohort study showed that most SJIA patients achieve inactive disease or disease remission when intervened early in the disease with recombinant IL-1 receptor antagonist (rIL-1RA, Anakinra) therapy [24,25,26,27]. These observations support the existence of a so-called “window of opportunity”. Apparently, at least in a significant subset of SJIA patients, the early phase of this disease is dominated by IL-1-dependent disease mechanisms, and early intervention by (first-line) therapeutic IL-1 blockade has resulted in excellent clinical outcomes [26,27]. The prospective Dutch cohort study using rIL-1RA therapy as a first line therapy in a treat-to-target approach reported excellent disease remission rates three to five years after diagnosis without the need for maintenance therapy in the majority of patients. The rates for chronic arthritis after three to five years are remarkably low, when compared to pre-rIL-1RA cohorts [26,28,29].

However, notwithstanding the intriguing response to IL-1 blockade early in the disease course, it should be recalled that not all SJIA patients respond equally well to rIL-1RA therapy [25,30,31]. In a sample of 22 patients with systemic-onset JIA treated with rIL-1Ra, Gattorno et al. identified two subsets of SJIA that can be identified according to the patient response to IL-1 blockade [31]. Ten patients with SJIA exhibited a dramatic response and were classified as complete responders. Eleven patients had an incomplete response or no response, and one patient could not be classified in terms of response. Compared with patients who had an incomplete response or no response, complete responders had a lower number of active joints and an increased absolute neutrophil count. In the study by Pascual et al. [32], eight of nine patients with systemic-onset JIA who showed good response to anakinra also had mild articular involvement, with a mean number of active joints and total white blood cell count that were very similar to those observed in the group of responder patients reported by Gattorno et al. Apparently, the targeted blockade of IL-1 by rIL-1RA is insufficient in a subset of patients to prevent or overcome a perpetuating loop of chronic inflammation [33]. 

### 4.2. The Example of a Recent Case Series: New Articular form of AOSD or Alternate Diagnosis of an Associated Inflammatory Joint Disease?

Supporting this possibility of phenotype change, we have recently reported eight Still’s disease patients (five adults, three children) who also presented features suggestive of spondyloarthritides (SpA) throughout their evolution [34]. All SJIA patients fulfilled the International League of Associations for Rheumatology (ILAR) criteria, all AOSD patients fulfilled the Yamaguchi and the Fautrel criteria, and all patients fulfilled either the Assessment of SpondyloArthritis international Society (ASAS) classification criteria for axial or peripheral spondyloarthritis, European Spondyloarthropathy Study Group (ESSG) criteria or Classification Criteria for Psoriatic Arthritis (CASPAR) criteria for psoriatic arthritis. In all patients but one, SpA manifestations occurred several years after SJIA/AOSD onset (mean delay 6.2 ± 3.8 years). Two patients had peripheral and three axial SpA, four developed psoriatic arthritis, and one SAPHO syndrome.

The articular manifestations of our patients were different from the classical rheumatic involvement described in AOSD recalled in Chapter 3.2. Besides the “classical” carpal ankylosis and narrowing of proximal and distal interphalangeal (PIP, PID) found in five patients, other articular manifestations were suggestive of SpA: uni- or bilateral sacroiliitis (n:3,); bilateral coxitis (n:1); erosion, ankylosis and reconstruction “PsA-like” signs on proximal and distal interphalangeal joints (n:2): multifocal osteitis and osteolytic bone lesions suggestive of SAPHO (n:1).

The association of both diseases in the same patients suggests common pathogenic pathways between SJIA/AOSD and SpA, and legitimately raises the question of an overlap, or more probably, a shift. The main cytokines produced during SJIA/AOSD flares are IL-1β, IL-18 and IL-6 [1,35]. Along with IL-23, these cytokines participate in the differentiation of Th17 cells [36]. These cells play a particularly important role in the peripheral form of SpA and psoriatic arthritis. An increase of IL-17 in SJIA/AOSD has also been reported [1,16], while it is the determining cytokine in the axial form of SpA [36]. One could imagine that the “cytokine burst” during an SJIA/AOSD flare up could trigger, through an overexpression of these cytokines, a form of SpA, and could thus be considered more a shift than an overlap. IL17 is involved in the recruitment of neutrophils, thus contributing to the maintenance of the inflammatory phenomenon [37]. This could partly explain the difficulty in treating some of these patients. Secukinumab, an anti-IL17 monoclonal antibody used for treating SpA, was necessary to reach remission in one patient. Interestingly, in this patient, AOSD was in complete remission (under anakinra, an anti-IL1 biological agent) at the onset of SpA manifestations.

Consistent with our findings and hypotheses, several works report the possibility of a switch of phenotype in autoinflammatory disorders, suggesting that autoinflammation may turn into “autoimmune inflammation” [33]. Prolonged elevation of IL-1β and IL-18 in patients with autonomous auto-inflammatory states can also affect T cell differentiation [38,39]. Auto-immune animal models show that IL-1signaling in T cells, when synergizing with IL-6 and IL-23 activation, results in the induction/differentiation of Th17 cells and to Th17-mediated immunopathology [40]. This Th17 polarization in IL-1-mediated disease has been shown to occur in familial Mediterranean fever (FMF) patients as well [41]. Interestingly, in FMF, up to 3% of the patients will eventually develop often HLA-B27-negative spondyloarthropathy, mainly sacroiliitis [42,43]. It is noteworthy that our patients were also all HLA-B27 negative.

### 4.3. AOSD as a SAID at the Crossroad of Other (Auto)Inflammatory Disorders and Points Left Unresolved

The pigeonholing of Still’s disease has been an issue since Bywaters first described the disease in adults [15]. The evolution towards an SpA in our patients opens up new avenues of reflection. The current perception of Still’s disease (SJIA and AOSD) tends to consider this disease as the archetype of non-familial or sporadic systemic autoinflammatory disorders [1]. Similarly, current insights argue for an autoinflammatory rather than autoimmune origin in SpA [44]. In fact, since the mid-2000s, the classification of immune-mediated inflammatory disorders has been refined, and rather than a two-tiered classification, with autoimmunity on one side and autoinflammation on the other—which might be applied for some rare monogenic autoimmune or autoinflammatory diseases—in reality, these categories represent a continuum [45,46,47]. Hence, beside the “classical” extra-articular manifestations of SpA, such as uveitis, skin psoriasis or inflammatory bowel disease, which can actually be regarded as associated diseases [48], other associations of autoinflammatory patients have been described, for instance, SpA and Behçet’s disease [49], SpA and Sarcoidosis [50], SpA and hidradenitis [51], pyoderma gangrenosum and inflammatory bowel diseases or inflammatory arthritides [52].

**Figure 2:**
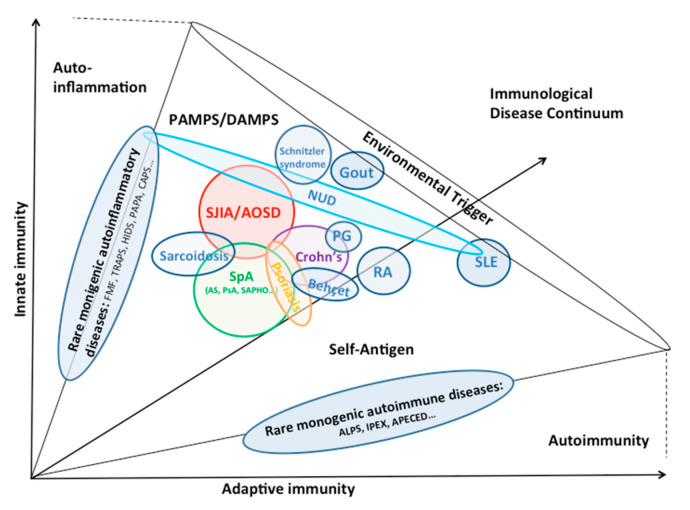
Continuum between AOSD and other autoinflammatory disorders. Diseases of the immune system are classified according to whether the innate immune system (myeloid lineage) is mainly responsible for the disease (autoinflammation) or the adaptive immune system (lymphoid lineage, autoimmunity). A disease spectrum includes rare monogenic diseases at the polar ends of the spectrum, and polygenic diseases, involving both myeloid and lymphoid cells in pathogenesis, in the center. This diagram adds a third variable, environmental triggers, to further define the pathogenesis of these diseases. The figure does not include all immunological recognized diseases, because of their large number, but focuses on the continuum between AOSD and other polygenic autoinflammatory disorders (please note the overlap among certain conditions). It is adapted from [45,46,47]. AOSD and SJIA are currently considered to be the same disease occurring at different moments of life. ALPS, autoimmune lymphoproliferative syndrome; AOSD, adult-onset Still’s disease; APECED, autoimmune polyendocrinopathy—candidiasis—ectodermal—dystrophy syndrome; AS, ankylosing spondylitis; CAPS, cryopyrin-associated periodic syndrome; DAMPS, danger-associated molecular patterns; FMF, familial Mediterranean fever; HIDS, hyperimmunoglobulinaemia D with periodic fever syndrome; IPEX, immune dysregulation polyendocrinopathy enteropathy X-linked syndrome; NUD, neutrophilic urticarial dermatosis; PAMPS, pathogen-associated molecular patterns; PAPA, pyogenic arthritis-pyoderma gangrenosum-acne syndrome; PG, pyoderma gangrenosum; PsA, psoriatic arthritis; RA, rheumatoid arthritis; SAPHO, synovitis acnea pustulosis hyperostosis osteitis syndrome; SLE, systemic erythematosus lupus; SpA, spondyloarthritis (include ankylosing spondylitis, psoriatic arthritis, reactive arthritis, seronegative arthritis associated with inflammatory bowel diseases and SAPHO syndrome); TRAPS, tumour necrosis factor receptor-associated periodic syndrome.

Similarly, an association between AOSD and other inflammatory diseases has been reported: Crohn’s disease [53,54,55], sarcoidosis [56,57] and neutrophilic urticarial dermatosis (NUD) [58,59]. The latter is a rare form of dermatosis with clinical and biological features very similar to AOSD (high fever, skin eruption, arthralgia, biological inflammation, neutrophilic hyperleukocytosis). Although it may be isolated, NUD often occurs in a setting of underlying systemic disease. The most commonly associated diseases are adult-onset Still’s disease, Schnitzler syndrome, systemic erythematosus lupus and cryopyrin-associated periodic syndromes (CAPS). Interestingly, if NUD shares common clinical and biological features with AOSD, the treatment may also be based on the same principle, with a notably very good response to anti-IL1 agents [59]. All these observations suggest a common pathophysiological background and pathways between AOSD and other inflammatory diseases, which need to be clarified: involvement of innate immune cells, especially neutrophils in AOSD [1,17], NUD [58,59] and SpA [37]; the central role of the NLRP3 inflammasome leading to caspase activation and overproduction of active IL-1β in AOSD, NUD and Schnitzler syndrome [1,17,59,60]; involvement of cytokines such as IL-18 and IL-6 in AOSD and Crohn’s disease [1,16,17,61,62] or IL-17 in AOSD and SpA [1,36], etc. Figure 2 shows the continuum between AOSD and the other polygenic autoinflammatory disorders. It opens the door to new possible phenotypes depending on the evolution of each patient. Whether the transition from one condition to another corresponds to an overlap or a shift remains unresolved. In other words, are we in front of two trains using the same track, or one train using multiple tracks?

However, clinicians should keep in mind that an atypical form of AOSD is likely to be another entity inside the large SAID group. These atypical “Still-like” entities are likely to be reclassified in the near future thanks to progress in research, particularly in genetics and molecular biology, as evidenced by the recent discovery of VEXAS syndrome, which enabled the reclassification of patients formerly diagnosed as “atypical” AOSD as VEXAS [63]. The same applies to the less recent discovery of Schnitzler syndrome, which is strikingly very close to AOSD, the main difference being the presence of a monoclonal immunoglobulin M component [64].

## 5. Conclusions

The classification of autoinflammatory diseases in general, and AOSD in particular, is a long-term task that has only just begun.

AOSD remains a complex and certainly heterogeneous disease. Its evolution modes are always difficult to forecast, so such patients must be cared for in reference centers for rare diseases, which are now connected internationally within the European Reference Network for the European Network on Rare Immunodeficiency, auToinflammatory and Auto-immune diseases (http://rita.ern-net.eu/) (accessed date 3 June 2021). There is a crucial need for better understanding of AOSD pathogenesis and more specific molecular diagnosis and probably for its disentanglement in several more specific entities in terms of mechanisms, clinical expression, or evolution. Prospective disease-specific cohorts and international collaborative registries are thus required to better stratify the different clinical phenotypes and possible continuum with other autoinflammatory disorders. In addition, the future research agenda should correlate clinical phenotypes with cytokine profiles and possibly with genotypes.

## Figures and Tables

**Table 1:** Possible manifestations of AOSD.

**Cardinal Manifestations**
Skin rash
Fever > 39 °C
Leukocytes > 10,000/mm^3^, neutrophils > 80%
Arthritis and arthralgia
**Other frequent manifestations**
Odynophagia, pharyngitis
Myalgia, myositis
Lymphadenopathy, splenomegaly
Hepatomegaly, hepatitis
Pericarditis, myocarditis, pleuritis, lung disease (interstitial lung infiltrates)
Increased ESR, CRP, fibrinogen
Increased ferritin, decreased glycosylated ferritin
**Life-threatening complications**
Tamponade, myocarditis, and acute respiratory syndrome
Pulmonary arterial hypertension
Fulminant hepatitis
Macrophage activation syndrome
Disseminated intravascular coagulopathy
Thrombotic microangiopathy

CRP; C-reactive protein; ESR, erythrocyte sedimentation rate.

**Table 2:** Classification criteria for AOSD.

Criteria	Yamaguchi et al. [4]	Fautrel et al. [5]
Major criteria	Fever ≥ 39 °C lasting one week or more Arthralgia lasting two weeks or more Typical skin rash: maculopapular, non-pruritic, salmon-pink rash with concomitant fever spikes Leukocytosis ≥ 10,000/mm^3^ with neutrophil polymorphonuclear proportion ≥ 80%	Spiking fever ≥ 39 °C Arthralgia Transient erythema Pharyngitis Neutrophil polymorphonuclear proportion ≥ 80% GF proportion ≤ 20%
Minor criteria	Pharyngitis or sore throat Lymphadenopathy and/or splenomegaly Liver enzyme abnormalities (aminotransferases) Negative for RF or antinuclear antibodies	Typical rash Leukocytosis ≥ 10,000/mm^3^
Exclusion criteria	Absence of infection, especially sepsis and Epstein–Barr viral infection Absence of malignant diseases, especially Lymphomas Absence of inflammatory disease, especially polyarteritis nodosa	None
Criteria requirement	At least five criteria, including two major criteria and no exclusion criteria	Four major criteria or three major criteria and two minor criteria
Set performance	Sensitivity 96.3%, specificity 98.2%, PPV 94.6% and NPV 99.3% Modified Yamaguchi criteria, i.e., Yamaguchi criteria and ferritin > ULN: sensitivity 100%, specificity 97.1%, PPV 87.1% and NPV 100% Alternative modified Yamaguchi criteria, i.e., Yamaguchi criteria and GF ≤ 20%: sensitivity 98.2%, specificity 98.6%, PPV 93.0% and NPV 99.6% [13]	Sensitivity 87.0%, Specificity 97.8%, PPV 88.7% and NPV 97.5% [13]

AOSD, adult-onset Still’s disease; GF, glycosylated ferritin; NPV, negative predictive value; PPV, positive predictive value; RF, rheumatoid factor; ULN, upper limit of normal.
